# Low Molecular Weight Heparin Nebulization Attenuates Acute Lung Injury

**DOI:** 10.1155/2017/3169179

**Published:** 2017-05-15

**Authors:** Nianlin Xie, Menglei Huan, Feng Tian, Zhongping Gu, Xiaofei Li

**Affiliations:** ^1^Department of Thoracic Surgery, Tangdu Hospital, The Fourth Military Medical University, Xi'an 710038, China; ^2^Department of Pharmaceutics, School of Pharmacy, The Fourth Military Medical University, Xi'an 710032, China

## Abstract

**Background:**

As acute lung injury (ALI) caused high mortality rate, it is important to explore the protection and treatment of ALI. The aim of the current study is to evaluate the effect of low molecular weight heparin (LMWH) nebulization on attenuating acute lung injury and the associated mechanism.

**Methods:**

The arterial blood gas, total protein content in bronchoalveolar lavage fluid, lung wet/dry weight ratio, malondialdehyde (MDA) content, superoxide dismutase (SOD) and glutathione peroxidase (GSH-Px) activity, and Akt phosphorylation were evaluated after the ALI rabbits were treated with or without LMWH nebulization.

**Results:**

PaO_2_ was increased and lung wet/dry weight ratio as well as total protein content in BALF was decreased after LMWH nebulization. After the application of LMWH nebulization therapy, the SOD and GSH-Px activity was rebounded and the increase of MDA content was significantly inhibited. The Akt protein phosphorylation level was decreased after LMWH nebulization therapy.

**Conclusions:**

LMWH nebulization treatment can relieve the traumatic ALI in rabbits and inhibit oxidative stress possibly by suppressing the Akt phosphorylation.

## 1. Introduction

Acute lung injury (ALI) is caused by a variety of lung damaging disease. Refractory hypoxemia and respiratory distress are the main clinical features of ALI. Many studies have reported that ALI has a mortality rate of 40 to 60 percent [1]. Due to its high mortality rate, exploring the protection and treatment of ALI is getting more and more attention of the people. It is reported that ALI is a set of clinical syndromes with pathological basis of increased vascular permeability, fibrin deposition, and a large number of edema fluid accumulations inside alveoli [2]. ALI is characterized of inflammatory mediator releasing, blood coagulation, and fibrinolysis dysfunction [3, 4].

As is known to all, heparin is an important anticoagulant and has already been widely used as anticoagulant agent clinically. The anticoagulant effect of heparin in vivo or in vitro is very strong. In addition, heparin can inhibit inflammation, improve microcirculation, regulate blood coagulation disorders, and may therefore possibly have a certain therapeutic effect against ALI. By contrast, low molecular weight heparin (LMWH) is a kind of heparin produced by nitrous acid decomposition and purification of low molecular heparin sodium or calcium salts. LMWH has the function of fast and continuous thrombosis resistance and can improve hemodynamics. It has higher bioavailability, longer half-life, and lower bleeding risk than ordinary heparin so it is more secure and easier to use [5].

In recent years, more and more research focused on heparin therapy on ALI. Han et al. reported that unfractionated heparin may attenuate endotoxin-induced lung vascular leak in ALI model of mice [6]. Mu et al. noted that heparin significantly ameliorated the lung injury induced by LPS in rats and may be a potential therapeutic reagent for treating ALI in the future [7]. Wang et al. found that the anti-inflammatory mechanisms of heparin in ALI may alleviate the inflammatory reaction in rabbits [8].

Although the previous studies discussed and put forward the effect of heparin in the treatment of ALI, they adopted the heparin intravenously. There is a possibility that intravenous administration may reduce the local function of heparin and cause the whole body adverse effects such as systemic complications. In order to avoid these problems, we propose to study the effects of LMWH by local nebulization delivery in the treatment of ALI in the current study.

In order to explore the therapeutic effects and the related mechanism of LMWH nebulization on ALI, we observed the arterial blood gas, total protein content in bronchoalveolar lavage fluid, lung wet/dry weight ratio, malondialdehyde (MDA) content, superoxide dismutase (SOD) and glutathione peroxidase (GSH-Px) activity, and Akt phosphorylation after establishing the model of ALI rabbits.

## 2. Materials and Methods

### 2.1. ALI Model

Before the induction of ALI, rabbits were fasted overnight but allowed water ad libitum. Animals in experimental groups were anesthetized with intraperitoneal pentobarbital (50 mg/kg) and were impacted on the right chest with driving-velocity at the following parameters: velocity 20 m/s, compression 20%, and weight 5.7 kg to develop ALI model. All animal protocols were approved by the Committee on Animal Investigations of Fourth Military Medical University.

### 2.2. Treatment Groups

Twenty-four healthy male New Zealand white rabbits (supplied by the Experimental Animal Center of Fourth Military Medical University, Xi'an, Shaanxi, China), weighing 2.5 to 3.2 kg, were divided into three groups at random (*n* = 8 each): control group in which animals were exposed to room air in the same cabinet; saline nebulization group in which animals were repetitively administered with saline solution (immediately after being impacted on the right chest and subsequently every 4 h thereafter) by ultrasonic nebulization via a tightly fitting mask and under spontaneous breathing; and low molecular weight heparin nebulization group in which animals were repetitively administered with low molecular weight heparin (1 U/g, immediately after being impacted on the right chest and subsequently every 4 h thereafter) by ultrasonic nebulization via a tightly fitting mask and under spontaneous breathing.

### 2.3. Arterial Blood Gas Analysis

Arterial blood was collected at 24 hours after ultrasonic nebulization for arterial blood gas analysis.

### 2.4. Total Protein Content in Bronchoalveolar Lavage Fluid

Animals were anesthetized with intraperitoneal pentobarbital (50 mg/kg). A median sternotomy allowed for exposure of both of the lungs. The trachea was exposed and inserted with an intravenous infusion needle. After ligating the hilum of the right lung, the left lung was lavaged 5 times with 0.5 mL ice-cold phosphate buffered saline. The recovery ratio of the fluid was about 90%. The bronchoalveolar lavage fluid (BALF) was immediately centrifuged at 500*g* for 10 min at 4°C, and the cell-free supernatant was stored at –80°C for analysis of the content of total protein in BALF.

### 2.5. Lung Wet/Dry Weight Ratio

As an index of lung edema, the amount of extravascular lung water was calculated. The middle lobe of the right lung was excised and the wet weight was recorded. The lobe was then placed in an incubator at 80°C for 24 h to obtain the dry weight. And the wet/dry weight ratios were calculated by dividing the wet weight by the dry weight.

### 2.6. Malondialdehyde (MDA) Content as Well as Superoxide Dismutase (SOD) and Glutathione Peroxidase (GSH-Px) Activity Assay

The lungs were homogenized in ice-cold phosphate buffered saline and centrifuged at 500*g* for 10 min at 4°C. The supernatant was stored at –80°C for analysis of MDA content and activity of SOD and GSH-Px with test kits (Nanjing Jiancheng Bioengineering Institute, Nanjing, Jiangsu, China) according to kit instructions.

### 2.7. Immunoblot Analysis

The lungs were homogenized in lysis buffer (20 mM HEPES pH 7.4, 1% Triton X-100, 10% glycerol, 2 mM ethylene glycol-bis (b-aminoethyl ether)-N,N,N′,N′-tetraacetic acid, 50 *μ*M b-glycerophosphate, 1 mM sodium orthovanadate, 1 mM dithiothreitol, 400 *μ*M aprotinin, and 400 *μ*M phenylmethylsulfonyl fluoride), transferred to Eppendorff tubes, and placed on ice for 15 minutes. Tubes were centrifuged at 14,000 rpm for 10 minutes at 4°C and supernatant was flash frozen. Crude cell lysates were matched for protein concentration, resolved on a 10% bisacrylamide gel, and electrotransferred to Immobilon-P membranes (Millipore Corp., Bedford, MA, USA). For assay of Akt phosphorylation and Akt total protein expression, Western blot analyses were performed with antibodies of phospho-Akt and Akt (Cell Signaling Biotechnology, Beverly, MA, USA). Blots were developed by enhanced chemiluminescence (NEN Life Science Products, Boston, MA, USA).

### 2.8. Statistical Analysis

Data of each group were expressed as mean ± standard deviation (SD). Results were processed by variance analysis and *t*-test with the software of SPSS 13.0. Statistical significance was set at *P* < 0.05.

## 3. Results

### 3.1. Effects of LMWH Nebulization on Partial Pressure Arterial Oxygen (PaO_2_)

**Figure 1:**
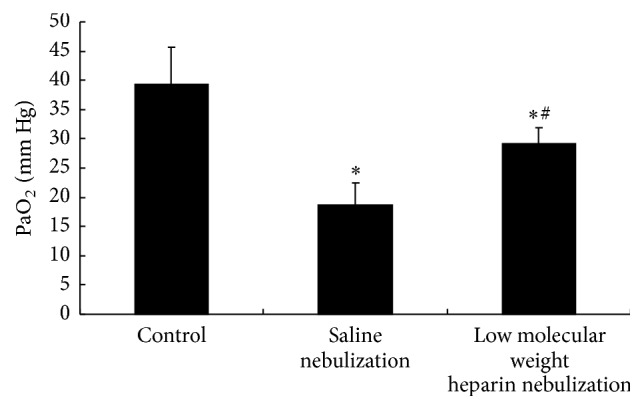
Effects of low molecular weight heparin nebulization on partial pressure arterial oxygen (PaO_2_). Arterial blood was collected at 24 hours after ultrasonic nebulization for arterial blood gas analysis. Data are presented as the mean ± SD. ^*∗*^*P* < 0.05 versus control group; ^#^*P* < 0.05 versus saline nebulization group.

In saline nebulization group, the partial pressure arterial oxygen (PaO_2_) was significantly decreased compared with control group (*P* < 0.05). However, low molecular weight heparin nebulization attenuated this decrease in PaO_2_ (Figure 1). Although PaO_2_ in LMWH nebulization group was still lower than that in control group (*P* < 0.05), PaO_2_ was significantly higher after LMWH nebulization than that in saline nebulization group (*P* < 0.05).

### 3.2. Effects of LMWH Nebulization on Total Protein Content in BALF

**Figure 2:**
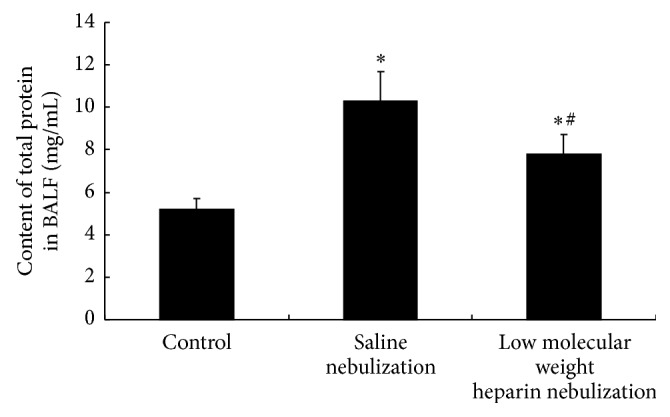
Effects of low molecular weight heparin nebulization on content of total protein in bronchoalveolar lavage fluid (BALF). After the animals were anesthetized, the left lung was lavaged 5 times with ice-cold phosphate buffered saline. The BALF was centrifuged and the supernatant was analyzed for the content of total protein in BALF. Data are presented as the mean ± SD. ^*∗*^*P* < 0.05 versus control group; ^#^*P* < 0.05 versus saline nebulization group.

Compared with control group, the content of total protein in BALF in both saline nebulization group and low molecular weight heparin nebulization group increased significantly (*P* < 0.05, Figure 2), while content of total protein in BALF in LMWH nebulization group decreased significantly compared to saline nebulization group (*P* < 0.05, Figure 2).

### 3.3. Effect of LMWH Nebulization on Lung Wet/Dry Weight Ratio

**Figure 3:**
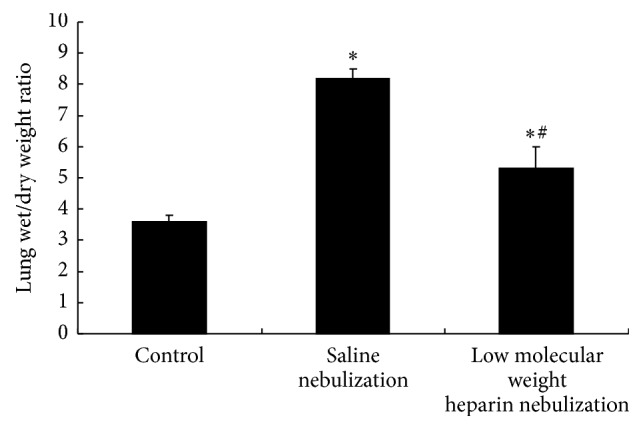
Effects of low molecular weight heparin nebulization on ratio of lung wet/dry weight. The middle lobe of the right lung was excised and the wet weight was recorded. The lobe was then placed in an incubator at 80°C to obtain the dry weight. The weight ratios were calculated by dividing the wet weight by the dry weight. Data are presented as the mean ± SD. ^*∗*^*P* < 0.05 versus control group; ^#^*P* < 0.05 versus saline nebulization group.

As shown in Figure 3, the lung wet/dry weight ratio in saline nebulization group was significantly increased compared with control group (*P* < 0.05). The increase in lung wet/dry weight ratio was significantly reduced by LMWH nebulization (*P* < 0.05).

### 3.4. Effects of LMWH Nebulization on MDA Content, SOD, and GSH-Px Activity

**Figure 4:**
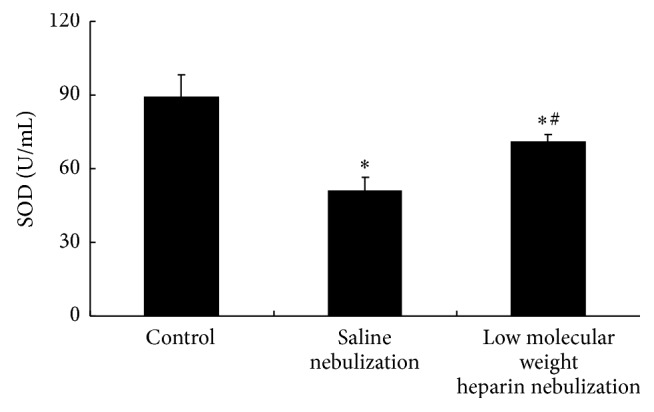
Effects of low molecular weight heparin nebulization on SOD activity. The lungs were homogenized in ice-cold phosphate buffered saline and centrifuged. The supernatant was collected to determine the activity of SOD. Data are presented as the mean ± SD. ^*∗*^*P* < 0.05 versus control group; ^#^*P* < 0.05 versus saline nebulization group.

**Figure 5:**
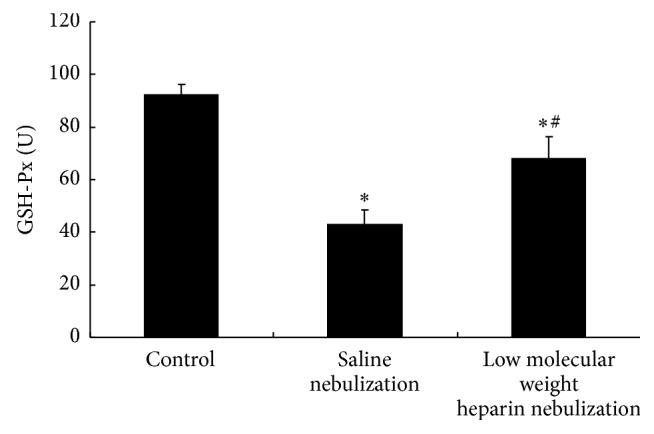
Effects of low molecular weight heparin nebulization on GSH-Px activity. The lungs were homogenized in ice-cold phosphate buffered saline and centrifuged. The supernatant was collected to determine the activity of GSH-Px. Data are presented as the mean ± SD. ^*∗*^*P* < 0.05 versus control group; ^#^*P* < 0.05 versus saline nebulization group.

**Figure 6:**
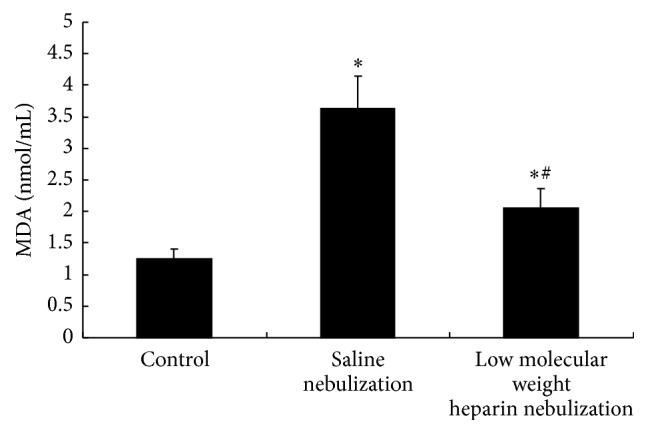
Effects of low molecular weight heparin nebulization on MDA content. The lungs were homogenized in ice-cold phosphate buffered saline and centrifuged. The supernatant was collected to determine the MDA content. Data are presented as the mean ± SD. ^*∗*^*P* < 0.05 versus control group; ^#^*P* < 0.05 versus saline nebulization group.

Compared with control group, activity of SOD and GSH-Px in saline nebulization group was significantly decreased (*P* < 0.05, Figures 4 and 5), and content of MDA in saline nebulization group was significantly increased (*P* < 0.05, Figure 6). In low molecular weight heparin nebulization group, activity of SOD and GSH-Px was significantly higher than that in saline nebulization group (*P* < 0.05, Figures 4 and 5), and content of MDA was significantly lower than that in saline nebulization group (*P* < 0.05, Figure 6).

### 3.5. Effects of LMWH Nebulization on Akt Phosphorylation

**Figure 7:**
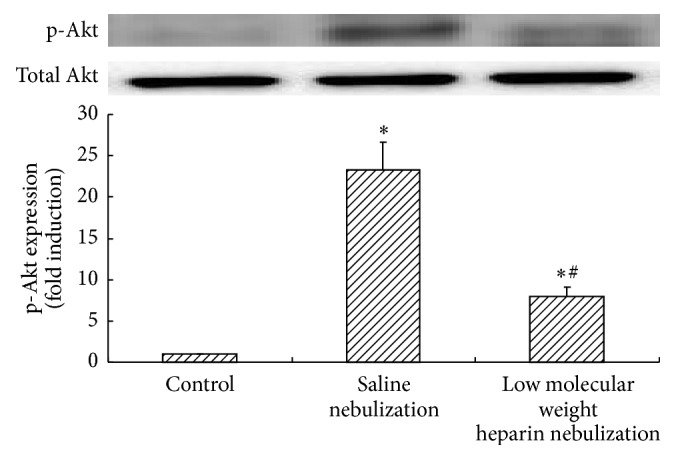
Effects of low molecular weight heparin nebulization on Akt phosphorylation. The lungs were homogenized in lysis buffer and centrifuged. The cell lysates were collected and the expression of Akt phosphorylation (p-Akt) and Akt total protein (Total Akt) was determined by Western blot. Level of p-Akt was quantitated by densitometry and plotted as fold induction. Data are presented as the mean ± SD. ^*∗*^*P* < 0.05 versus control group; ^#^*P* < 0.05 versus saline nebulization group.

Because the Akt phosphorylation is associated with oxidative stress response, we subsequently examined the effects of LMWH nebulization on Akt phosphorylation by ALI to explore the potential molecular mechanisms. The Akt phosphorylation on Ser473 was measured by Western blot and the data showed that the endogenous level of phospho-Akt expression (Ser473) in both saline nebulization group and LMWH nebulization group increased significantly (*P* < 0.05, Figure 7), while that in LMWH nebulization group was downregulated compared to that in saline nebulization group (*P* < 0.05, Figure 7). The ALI related Akt phosphorylation was attenuated by LMWH nebulization.

## 4. Discussion

As clotting disorder is one of the features of ALI, anticoagulants such as heparin may possibly have a certain therapeutic effect [9]. Our data showed that PaO_2_ was increased and lung wet/dry weight ratio as well as total protein content in BALF was decreased after the ALI rabbits were treated by LMWH nebulization, indicating that LMWH nebulization may improve pulmonary gas exchange, increase oxygenation index and decrease alveolar exudation, and therefore attenuate acute lung injury to some extent. Consistently, Li et al. found that lung wet/dry weight ratio in lung injury model of mice induced by high-tidal-volume ventilation was decreased by low-dose unfractionated heparin and enoxaparin, but they did not examine the effects of LMWH and the drugs were given subcutaneously [10]. We currently used LMWH by the dosing method of nebulization and also confirmed the therapeutic effects on ALI.

Furthermore, we tried to explore the underlying mechanism of the therapeutic effects of LMWH nebulization on ALI. It is reported that lung oxidative stress injury was aggravated in ALI [11–13]. Our results showed that the activity of SOD and GSH-Px was significantly decreased while the content of MDA was significantly increased in ALI, indicating that the traumatic ALI can lead to lung oxidative stress reaction. After the application of LMWH nebulization therapy, the SOD and GSH-Px activity was rebounded and the increase of MDA content was significantly inhibited, indicating that LMWH nebulization can play a role of antioxidant, reduce oxidative stress reaction, and attenuate the ALI. Rajeswari and Varalakshmi also found that LMWH has antioxidant potential in countering the oxidative challenge in the experimental oxidative renal injury model of rats [14].

Since oxidative stress can lead to the Akt phosphorylation [15, 16], it is important to explore whether the enhancement of oxidative stress caused by ALI in the current study is associated with the Akt phosphorylation. Lee et al. found that ALI caused by LPS can be alleviated through the inhibition of Akt phosphorylation in mice, indicating that the Akt phosphorylation has certain correlation with the severity of ALI [17]. Our results showed that phosphorylated Akt protein expression was increased in ALI, suggesting the Akt phosphorylation might be involved in the development of ALI. This was consistent with the changes of SOD, GSH-Px activity, and the MDA content detected in this study, indicating that the Akt phosphorylation is possibly involved in ALI related oxidative stress reaction.

As heparin was found to have antioxidant effects [18, 19] and the Akt phosphorylation was involved in oxidative stress, we therefore tried to detect whether LMWH has influence on the Akt phosphorylation. Li et al. found that LMWH administrated subcutaneously can reduce hyperoxia-augmented ventilator-induced ALI in mice through the inhibition of Akt phosphorylation [20]. Our current study found that Akt phosphorylation was restrained in ALI rabbits after LMWH nebulization therapy. At the same time, the changes of oxidative stress indicators were eased, indicating that LMWH nebulization might inhibit the Akt phosphorylation to reduce the levels of oxidative stress, so as to mitigate the effects of ALI and play a therapeutic role.

In conclusion, the present study found that LMWH nebulization treatment can relieve the traumatic ALI in rabbits, improve lung tissue activity of SOD and GSH-Px, reduce MDA content, and inhibit oxidative stress possibly by suppressing the Akt phosphorylation. This study discussed the effects of LMWH nebulization in the treatment of ALI with new delivery methods from the point of view of inhibiting oxidative stress. Also this study illustrated the related mechanism and offered experimental basis for the clinical treatment of ALI.
